# Hemp Protein Hydrolysates Modulate Inflammasome-Related Genes in Microglial Cells

**DOI:** 10.3390/biology12010049

**Published:** 2022-12-27

**Authors:** Sergio Montserrat-de la Paz, Gabriela Carrillo-Berdasco, Fernando Rivero-Pino, Alvaro Villanueva-Lazo, Maria C. Millan-Linares

**Affiliations:** 1 Department of Medical Biochemistry, Molecular Biology, and Immunology, School of Medicine, University of Seville, 41009 Seville, Spain; 2 Department of Food & Health, Instituto de la Grasa, CSIC, 41013 Seville, Spain

**Keywords:** neuroinflammation, microglia, inflammasome, hemp peptides, immunonutrition

## Abstract

**Simple Summary:**

Neuroinflammation can lead to the development of neurodegenerative diseases. Food-derived peptides released by the action of enzymes have been proven to modulate several physiological processes. In this study, peptides obtained from hemp protein were evaluated as anti-inflammatory agents employing a cell model, measuring the responses of inflammatory mediators, microglial polarization markers, and genes related to inflammasome activation, as markers of inflammation and the potential counteraction exerted by the peptides, related to neurodegenerative processes. Results showed a neuroprotective effect based on anti-inflammatory activity of the peptides, via the inflammasome. The use of these peptides in the diet could help to prevent inflammation and promote a healthy aging of humans.

**Abstract:**

A prolonged inflammatory response can lead to the development of neurodegenerative diseases such as Alzheimer’s disease. Enzymatic hydrolysis is a sustainable way to increase the value of protein sources by obtaining peptides that can exert bioactivity. Hemp (*Cannabis sativa* L.) protein hydrolysates have been proven to exert anti-inflammatory activity. In this study, two hemp protein hydrolysate (HPHs), obtained with Alcalase as sole catalyst, or with Alcalase followed by Flavourzyme, were evaluated as inflammatory mediators (TNFα, IL-1β, IL-6, and IL-10), microglial polarization markers (*Ccr7*, *iNos*, *Arg1*, and *Ym1*), and genes related to inflammasome activation (*Nlrp3*, *Asc*, *Casp1*, and *Il18*), employing the lipopolysaccharide (LPS)-induced neuroinflammation model in murine BV-2 microglial cells. A significant decrease of the expression of proinflammatory genes (e.g., *Tnfα*, *Ccr7*, *inos*, and *Nlrp3*, among others) and increase of the expression anti-inflammatory cytokines in microglial cells was observed after treatment with the test HPHs. This result in the cell model suggests a polarization toward an anti-inflammatory M2 phenotype. Our results show that the evaluated HPHs show potential neuroprotective activity in microglial cells via the inflammasome.

## 1. Introduction

Globalization has favored technological advances and the search for sustainability. At the same time, the food industry is aiming to develop products that meet consumers’ demands for functional foods that could prevent diseases [1]. In this regard, the interest over plant sources is increasing, with the current lifestyle changes of humans. In this line, hemp (*Cannabis sativa* L.) interest in the food industry is rapidly increasing currently because of its nutritional composition (e.g., protein content of whole hemp seed from 20% to 25%) [2,3] and because its production has been proven to be environmentally friendly in different aspects [4,5].

Hemp protein hydrolysates obtained by enzymatic hydrolysis have been proven to modulate diverse physiological processes [6], including in vitro anti-inflammatory properties, [7,8], antioxidant activities [9], and in vitro antidiabetic activity [10]. The functionality and efficacy of protein hydrolysates depends mainly on the peptide sequences (composition and length), which will vary depending on the enzymatic treatment employed. Studies regarding the mechanisms by which hemp protein-derived peptides may have an anti-inflammatory effect are so far limited. The focus of this paper is on the anti-inflammatory properties of hemp peptides.

Inflammation is a defensive response to pathogenic stimuli. As consequence, immune cells such as microglia, astrocytes, and activated T cells can trigger neuroinflammation. A persistent neuroinflammation can result the appearance of neurodegenerative diseases, such as Alzheimer’s. The capacity of the immune system to influence the central nervous system (CNS) is being studied as a potential therapy for the treatment of brain disorders (i.e., through immune modulation) [11]. Immune system cells, such as macrophages, in the stationary state, present an increased expression of molecular pattern recognition receptors (PRRs) [12]. After recognition of determined molecular patterns, these PRRs can modulate phagocytosis, promote a controlled response, and regulate gene expression (e.g., interferon-α/β, IL-1β, and tumor necrosis factor α (TNF-α)), to promote an innate response and establish an adaptive immune response [13,14].

Inflammasomes (e.g., NLRP3) are signaling complexes present in macrophages and directly related to the activation of PRRs, that regulate immune system cells, acting as sensors and mediators of inflammation [15,16]. Inflammasomes are initiated by activating Toll-like receptors (TLRs) and NOD-like receptors [12,13]. The activation of PRRs activates the transcriptional factor NF-κB, resulting in the synthesis of NLRP3, pro-IL-1β, pro-IL-18, and pro-IL-33 [12]. Consequently, controlling the subacute and chronic neuroinflammation processes can help to restore cellular homeostasis [17]. In this sense, blocking or inhibiting the activation of the NLRP3 inflammasome could improve neurological damage [18]. It has been recently shown, for instance, that early life stress, impacting immune system maturation, has an impact in modulating the response to a CNS damage [19]. As the evidence shows, the complexity of the mechanism behind these physiological processes is still to be unraveled.

It has been shown that food and health are interconnected since bioactive compounds can modulate certain physiological processes. MCC950 is known to be a specific inhibitor of the inflammasome, but the potential of food-derived peptides obtained by enzymatic hydrolysis has been scarcely studied. Bioactive peptides may inhibit oxidative stress and the inflammatory response in activated microglia, macrophage-like immune cells from the CNS, suggesting possible neuromodulatory therapeutic applications [9,14] without side effects. In this regard, the use of antioxidant hemp bioactive peptides could help minimize the economic impact of the neurodegenerative diseases’ treatment beyond the health consequences they have on the patient, while employing a protein source with nutritional interest and that is environmentally friendly. The unique amino acid composition and the peptides released after enzymatic hydrolysis show the potential of this vegetable source as anti-inflammatory agent. However, the metabolic response in cells is still to be investigated, especially in the microglia.

The aim of this work was to evaluate the potential anti-inflammatory activity exerted by two hemp protein hydrolysates obtained from industrial hemp (*Cannabis sativa* L.), and thus, to provide evidence on the effect, in terms of inflammation, that hydrolysates exert on microglia.

## 2. Materials and Methods

### 2.1. Chemical and Samples

*Cannabis sativa* L. seeds were provided by Sensi Seeds Bank. Alcalase 2.4 L and Flavourzyme (1000 L) were obtained from Novozymes (Bagsvaerd, Denmark). The murine BV-2 cell line was provided by the Cell Biology Unit (Instituto de la Grasa, IG-CSIC, Seville, Spain). Primers were purchased from Eurofins Biolab S.L.U (Barcelona, Spain). The total RNA was isolated using TRIsure (Bioline, Meridian Life Science, Inc. Memphis, TN, USA) and reverse-transcribed to cDNA using a Bio-Rad synthesis kit (Hercules, CA, USA). Quantitative PCR was performed using iTaq™ Universal SYBR^®^ Green Supermix from Bio-Rad. LPS (*E. coli* 055:B5) was purchased from Sigma-Chemical Co. (St. Louis, MO, USA). All the chemicals (reagents and solvents) were of analytical grade and provided by Sigma Chemical Co., Bachem AG (Bubendorf, Switzerland), and Gibco (Waltham, MA, USA).

### 2.2. Preparation of Hemp Protein Isolate

Hemp protein isolate (HPI—33.3% of protein) was obtained using the method of Lqari et al. [20] Briefly, hemp defatted flour was extracted using 0.25% Na_2_SO_3_ (*p/v*) at a pH of 10.5 for 1 h. After centrifuging the extract at 7500 rpm for 15 min, the supernatant was recovered, and the pellet was extracted again. Both supernatants were adjusted to the isoelectric point of hemp protein, and the precipitate was washed with distilled water adjusted to pH 4.3. Subsequently, it was centrifuged to remove residual salts and other non-protein compounds and the protein precipitate obtained was freeze-dried and stored at room temperature. HPI contains 96.5% of protein.

### 2.3. Hydrolysis of Hemp Protein Isolate

The hydrolysis was carried out under continuous stirring by diluting HPI in distilled water (10% *w/v*) at a temperature of 50 °C and pH 8. Two hemp protein hydrolysates (HPHs) were obtained: (i) HPH20A was obtained adding Alcalase (0.3 AU/g) for 20 min; (ii) HPH60A+15F was obtained adding Alcalase (0.3 AU/g) for 60 min; and then, at pH 7, Flavourzyme was added (60 LAPU/g) for 15 min [9]. Enzyme deactivation at the end of the hydrolysis was achieved by heating for 15 min at 85 °C. The chemical composition and amino acid characterization of hemp seeds and hemp protein products are shown in the Appendix A (Table A1 and Table A2). The choice of these specific hydrolysates is based on the antioxidant activity, as well as degree of hydrolysis and reaction time from previous works [9].

### 2.4. BV-2 Cell Culture and Treatments

Murine BV-2 cell lines were kept in a medium with a high glucose content (DMEM, Dulbecco’s modified Eagle medium), supplemented with 10% heat-inactivated fetal bovine serum and 1% penicillin/streptomycin. The CO_2_ level was 5% and temperature 37 °C in the incubator (Thermo Con Electron Corporation, Waltham, MA, USA). The experiments were carried out in 12-well plates. BV-2 microglial cells were seeded at a density of 5 × 10^5^ cells/well under different conditions. The samples tested included exposing cells to 1 μg/mL lipopolysaccharide (LPS) as a proinflammatory stimulus followed by the exposing of cells to different treatments (i) MCC950, as control, being a specific inhibitor of the inflammasome (I) (ii) HPH20A (iii) HPH60A+15F (iv) I + HPH20A, (v) I+HPH60A+15F; at a HPH concentration of 100 μg/mL. MCC950 was added at 10 μM and LPS was added 1 h before the treatment with protein hydrolysates. A negative control (untreated cells) and a positive control (cells exposed to 1 μg/mL LPS) were included. After 24 h of incubation, the cells were recovered to carry out the experiments.

### 2.5. Cell Viability Assay

Microglial BV-2 cells were incubated with different concentrations of HPHs in 96-well plates (10^5^ cells/well) for 24 h. The concentrations used were 10, 25, 50, 100, and 200 μg/mL. Then, the MTT solution was added and incubated until a purple precipitate was visible. The MTT crystals were solubilized with DMSO and subsequently measured using a microplate reader at 570 nm, corrected to 650 nm [21]. Cell survival is expressed as the percentage absorbance compared to that obtained for the untreated control cells. TRITON was used as the positive control for decreased cell viability.

### 2.6. Determination of NO in Supernatant

The supernatant obtained from the treated cells was transferred to a 96-well plate and mixed with Griess reagents. Then, it was incubated for 20 min at room temperature. The amount of nitrite was determined as an index of NO generation when extrapolated with a sodium nitrite standard curve, measuring the absorbance at 540 nm using a microplate reader [22].

### 2.7. RNA Isolation and Real-Time Quantitative PCR Analysis

RNA from BV-2 microglial cells was isolated to quantify gene expression using RT-qPCR. Total RNA was extracted using TRIsure Reagent (Bioline). The RNA quality was evaluated as a function of the A_260_/A_280_ ratio using a NanoDrop ND-1000 (ThermoFisher Scientific, Madrid, Spain). Briefly, the RNA (250 ng) underwent reverse transcription (iScript, BioRad), and 10 ng of cDNA was used as a template for RT-qPCR amplification. The mRNA levels for specific genes were determined using the CFX96 (Bio-Rad) system. For each PCR, we added the cDNA template containing the primer pairs for the genes or for glyceraldehyde 3-phosphate dehydrogenase (*Gapdh*) and hypoxanthine phosphoribosyltransferase (*Hprt*), used as housekeeping genes. All the amplification reactions were performed in triplicate, and the average threshold cycle (Ct) numbers were used to calculate the relative mRNA expression of the candidate genes. The magnitude of the change in mRNA expression of the candidate genes was calculated using the 2^−ΔΔCt^ standard method. All the data were normalized with respect to the content of both endogenous reference gene and expressed relative to the control values. The sequences of the engineered oligonucleotides are shown in Table 1.

### 2.8. Determination of Protein Levels by Enzyme-Linked Immunosorbent Assay (ELISA)

The quantification of the IL-10, IL-6, IL-1β, and TNF-α secreted by BV-2 cells into the medium after stimulation with LPS and subsequent treatment with HPHs and MCC950 was performed by enzyme-linked immunosorbent assay (ELISA) using kits purchased from Peprotech and following the specific protocol for each protein indicated by the manufacturer.

### 2.9. Statistical Analysis

The data were evaluated using Graph Pad Prism version 9.1.2 (San Diego, CA, USA). The statistical significance of any difference in parameters between the groups was assessed using two-way analysis of variance (ANOVA), followed by Tukey’s multiple-comparison test. Values are presented as the means ± SDs (*n* = 3); those marked with different letters are significantly different (*p* < 0.05).

## 3. Results

### 3.1. Evaluation of the Cytotoxicity of Hemp Protein Hydrolysates in BV2 Cells

**Figure 1:**
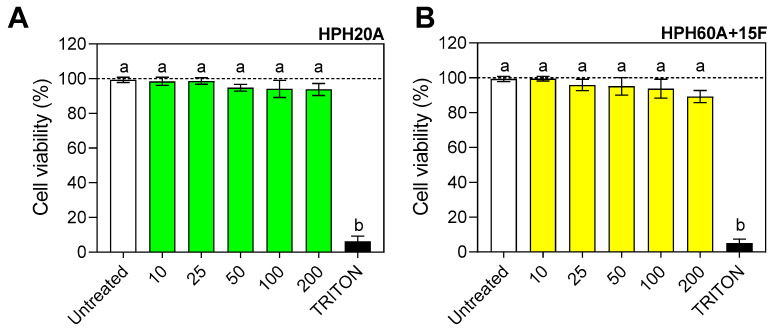
Cell viability (%) of BV-2 cells incubated for 24 h with HPH20A (**A**) and HPH60A+15F (**B**) (up to 200 µg/mL). Values are presented as the means ± SDs (*n* = 3); different letters denote significant differences among the samples. (*p* < 0.05). Control: TRITON.

The results (Figure 1) showed that both hydrolysates, HPH20A (Figure 1A) and HPH60A+15F (Figure 1B), used at concentrations ranging from 10 to 200 μg/mL, did not have an impact on cell viability, which was maintained at values around 95% for all the doses tested. When cells were treated with TRITON (control), cell viability was reduced around 10%.

### 3.2. Determination of the Antioxidant Activity of Hemp Products

**Figure 2:**
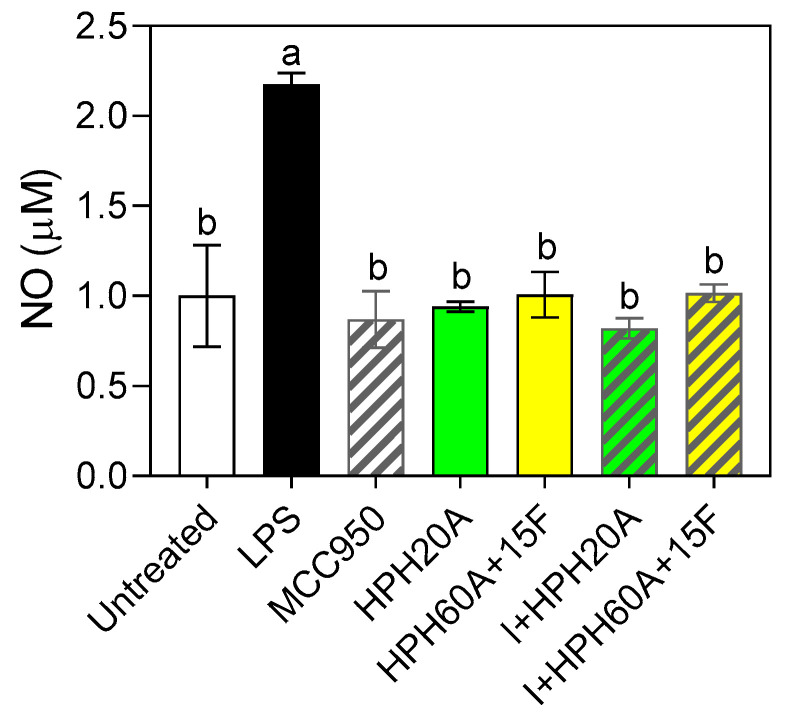
Determination of NO release in BV-2 cells with or without LPS stimulation (1 µg/mL) and subsequent incubation with MCC950 (I) and HPHs (100 µg/mL) for 24 h. Values are presented as the means ± SDs (*n* = 3); Different letters denote significant differences among the samples. (*p* < 0.05).

The stimulation of the BV-2 cells with LPS induced a 70% increase in NO release relative to the control without LPS (Figure 2), indicating that the inflammation occurred in the cells. However, when cells were subsequently treated with the HPHs (either with or without MCC950) a statistically significant decrease in NO release with respect to the control was reported, showing the antioxidant activity exerted by the samples under assessment in the cell model. No significant differences were found among all the samples tested. These results indicate that the peptides released after the enzymatic hydrolysis possess at least the same effect on NO release.

### 3.3. Neuroprotective Properties of Hemp Protein Hydrolysates in BV-2 Cells

**Figure 3:**
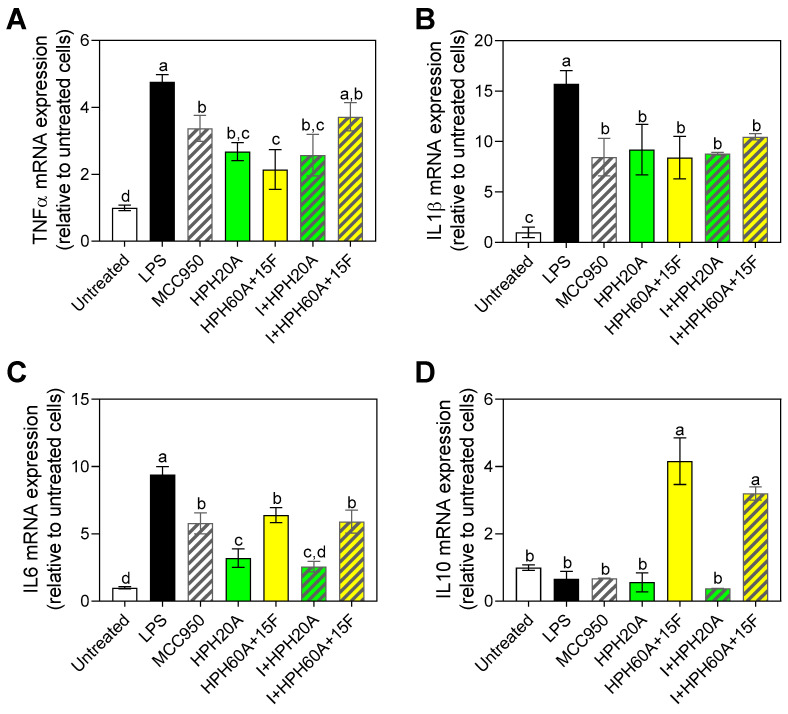
Gene expression of cytokines *Tnfα* (**A**), *Il1β* (**B**), *Il6* (**C**), and *Il10* (**D**) in BV-2 microglia cells after 24 h incubation with or without stimulation with LPS (1 μg/mL), as well as following treatment with MCC950 (I), a specific inhibitor of the inflammasome, and/or with HPHs (100 μg/mL). Values are presented as the means ± SDs (*n* = 3); Different letters denote significant differences among the samples. (*p* < 0.05).

The anti-inflammatory effect of protein hydrolysates at the brain level was evaluated in a cell culture model, by measuring the gene expression of the cytokines *Tnfα*, *Il1β*, *Il6*, and *Il10*. As shown in Figure 3, all the treatments analyzed led to a significant decrease of the expression of *Tnfα* (Figure 3A) with respect to the control. The largest decrease was observed in the sample employing the hydrolysate obtained with Alcalase and Flavourzyme (HPH60A+15F), significantly different from the cells treated only with the specific inhibitor MCC950. However, I+HPH60A+15F had a reduced ability to suppress the increase in TNF alpha mRNA expression compared to I or HPH60A+15F alone. Further investigations are needed to fully understand the mode of action of the HPHs. It has been shown that bioactivity of hydrolysates is due to the specific interaction of peptides with target receptors or enzymes. Consequently, the amount of bioactive sequences in the hydrolysates could explain the results hereby reported.

As shown in Figure 3B, all the treatments used repressed the gene expression of IL-1β with respect to the control, with no significant differences. The mechanism by which the hydrolysate and the inhibitor exerted the activity over the expression of different genes do not necessarily have to show a correlation, but these results offer an insight of how these samples can have an impact on cells at different levels. In Figure 3C, it is shown how all the treatments decreased the mRNA expression of IL-6 with respect to LPS-treated cells. However, this gene repression was higher following HPH20A and I + HPH20A treatments, showing a significant difference to the effect of MCC950, potentially indicating that the hydrolysate obtained using Alcalase (HPH20A) may not only act through the inflammasome pathway.

Only treatments including the hydrolysate HPH60A+15F, whether alone or administered with MCC950, increased the expression of IL-10 (Figure 3D), suggesting an anti-inflammatory effect of HPH60A+15F, whereas the other samples were not significantly different. The differences observed in the expression of different cytokines provided an insight on how the different hydrolysates can exert an effect by a specific mechanism.

**Figure 4:**
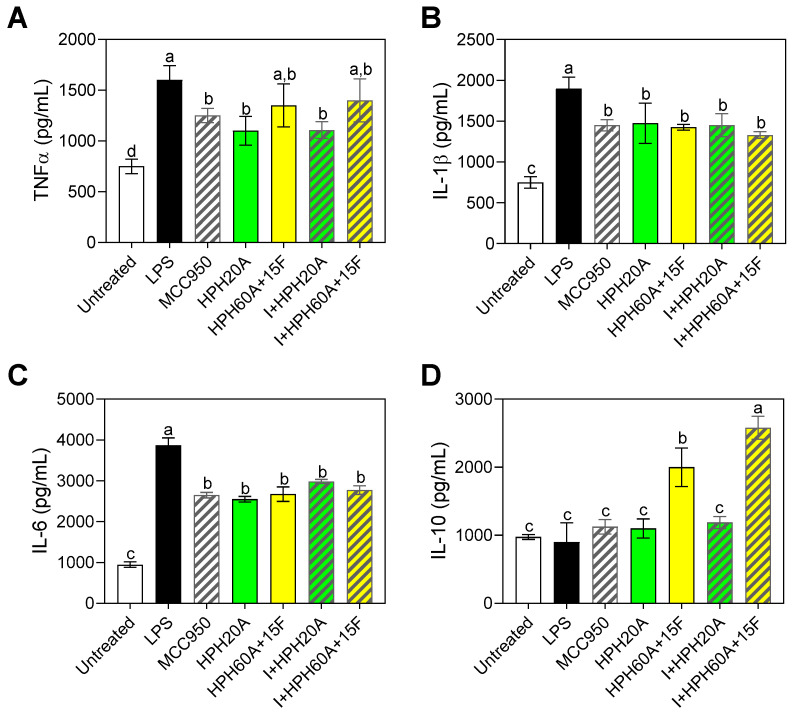
Secretion of TNF-α (**A**), IL-1β (**B**), IL-6 (**C**), and IL-10 (**D**) in BV-2 cells after 24 h incubation with or without stimulation with LPS (1 μg/mL), as well as following treatment with MCC950 (I), a specific inhibitor of the inflammasome, and/or with HPHs (100 μg/mL). Values are presented as the means ± SDs (*n* = 3); Different letters denote significant differences among the samples. (*p* < 0.05).

Figure 4 shows the results obtained by ELISAs for the cytokines TNF-α (Figure 4A), IL-1β (Figure 4B), IL-6 (Figure 4C), and IL-10 (Figure 4D). In all cases, the protein levels (pg/mL) after 24 h reflected the changes observed previously by RT-qPCR, increasing the evidence that the peptides contained in the hydrolysates are capable of modulating the immune response in the cells in vitro. Recent studies have also shown that protein hydrolysates from legumes have a potential anti-inflammatory effect by strongly suppressing proinflammatory gene expression induced by LPS, suggesting their potential to be used as ingredients in functional foods with immunomodulatory activity [23,24].

### 3.4. Effect of Hemp Protein Hydrolysates on Microglial Polarization

**Figure 5:**
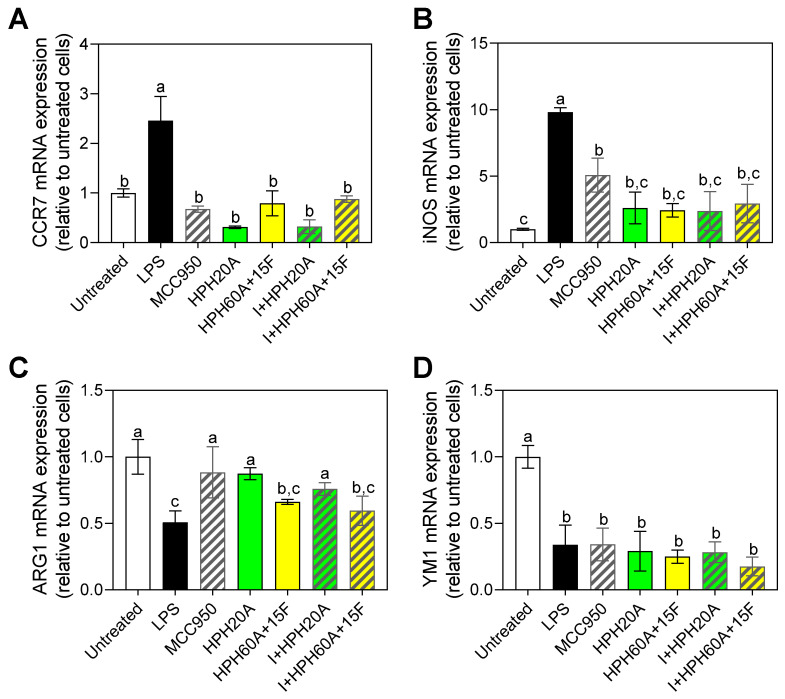
Gene expression of markers of polarization toward phenotypes M1, *Ccr7* (**A**) and *iNos* (**B**), and M2, *Arg1* (**C**) and *Ym1* (**D**) in BV-2 cells after 24 h incubation with or without stimulation with LPS (1 µg/mL), as well as following treatment with MCC950 (I) and/or HPHs (100 µg/mL). Values are presented as the means ± SDs (*n* = 3); Different letters denote significant differences among the samples. (*p* < 0.05).

As shown in Figure 5, *Ccr7* gene expression was significantly increased by LPS with respect to the untreated control (Figure 5A), while the gene expression of this cytokine when cells were subjected to the treatments significantly decreased with respect to the control with LPS. No significant differences were observed among the different samples, reaching the baseline levels of the control without LPS (negative control).

In Figure 5B, gene expression of *iNos* is shown. A significant increase in the gene expression of *iNos* was observed in the positive control (treatment only with LPS). In the cells exposed to the samples, the gene expression was decreased compared to the positive control. Around 50% of the expression was diminished in the MCC950, and a larger decrease was observed when cells were exposed to the HPHs, without significant differences among different treatments.

In the case of the M2 microglia polarization markers, as shown in Figure 5C, a significant increase in *Arg1* following treatment with MCC950 and HPH20A was observed with respect to cells treated with LPS. On the other hand, *Ym1* gene expression significantly decreased after stimulation with LPS with respect to the untreated control (Figure 5D), but there was no significant increase in *Ym1* mRNA levels following any of the treatments. This implies that HPHs do not have an in vitro effect over the expression of that marker, but instead the effect on the response depends on the gene expression modulation of other markers. The complexity behind the neuroinflammation response is still to be unraveled, in terms of physiological effects in humans and the response of bioactive compounds intake. These results provide an insight into the potential mechanisms behind the modulation of this inflammation.

### 3.5. Effect of Hemp Protein Products on Components of Inflammasome

**Figure 6:**
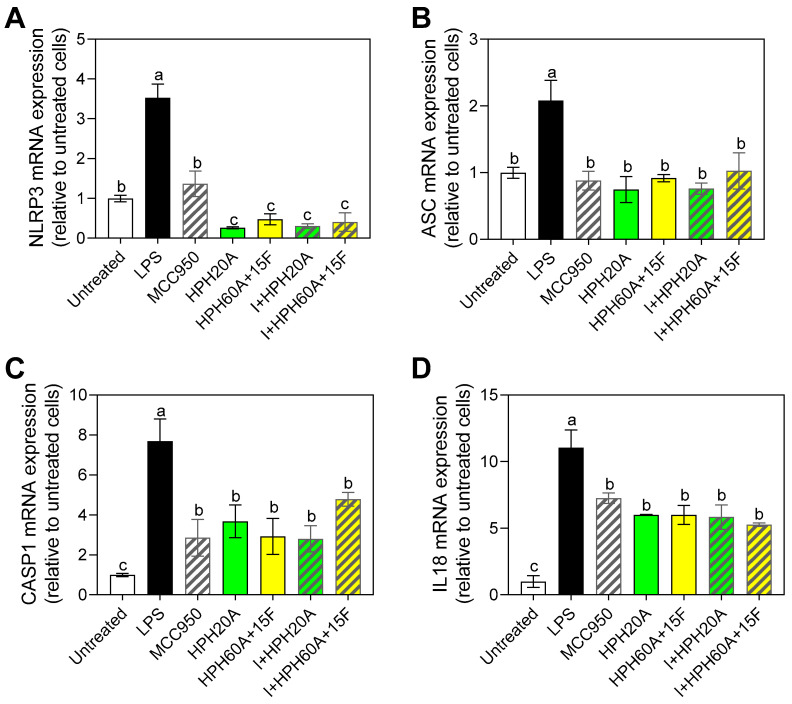
*Nlrp3* (**A**), *Asc* (**B**), *Casp1* (**C**), and *Il18* (**D**) mRNA gene expression in BV-2 cells after 24 h incubation with or without stimulation with LPS (1 μg/mL), as well as following treatment with MCC950 (I) as a specific inhibitor of the inflammasome and/or HPHs (100 μg/mL). Values are presented as the means ± SDs (*n* = 3); Different letters denote significant differences among the samples. (*p* < 0.05).

As shown in Figure 6, exposure to LPS caused a significant increase in the mRNA expression of *Nlrp3* (Figure 6A) with respect to the untreated control, while the subsequent exposure to protein hydrolysates individually or in combination significantly counteracted this LPS-induced change, thereby blocking the increased expression of *Nlrp3*. The decrease was significantly higher for the treatments involving hydrolysates than the individual administration of MCC950, again indicating the neuroprotective activity of these protein hydrolysates via the inhibition of inflammasome components, as well as through other pathways such as NF-κB [25]. Exposure to LPS caused a significant increase in the mRNA expression of *Asc* with respect to the untreated control (Figure 6B). Furthermore, as observed for *Nlrp3*, both hydrolysates negatively regulated the increase in *Asc* expression with respect to the control, with no significant differences between the treatments. These results overall suggest that there are different peptides in the pool composing the hydrolysate that can exert this anti-inflammatory activity and that the release of peptides after the Flavourzyme treatment did not ameliorate this effect. Instead, peptides released from the Alcalase-hydrolsis are responsible of these effects. A peptidome characterization is needed in order to clarify exactly which sequences are responsible of these effects.

In order to evaluate the effects of the modulation of this inflammasome, *Casp1* (Figure 6C) and *Il18* (Figure 6D) mRNA levels were determined. As shown in Figure 6C, in line with the results observed for *Nlrp3* and *Asc*, exposure to LPS caused a significant increase in *Casp1* expression with respect to the untreated control, whereas all the treatments considered in this study significantly counteracted this increase with respect to the LPS control. Finally, as seen for the other proinflammatory cytokines involved in the inflammasome, all the treatments also repressed the gene expression of *Il18* with respect to the control with LPS. No significant differences were found among treatments, in contrast to the effects observed for *Nlrp3*.

## 4. Discussion

Food proteins contain bioactive peptides that can be used to treat or prevent a number of diseases, including diabetes, thrombosis, and/or inflammation. There is a great deal of research on the bioactivity of protein hydrolysates, particularly in vitro experiments, employing traditional food sources (e.g., milk or fish proteins). However, there is scarce literature published employing vegetable proteins (only with commonly used ones such as soy), considered a more sustainable source than animal-derived proteins. In addition, these sources are considered a healthier alternative in terms of nutrition. In spite of that, the research on their potential as source of bioactive peptides is scarce, especially with alternative proteins such as hemp. In this study, the purpose was to investigate the efficacy of hemp peptides to manage neuroinflammation in a murine BV-2 microglial cells. The hydrolysates employed in this research were chosen among ten hydrolysates, as the most antioxidant ones, as described elsewhere [9].

None of the tested HPHs were cytotoxic to murine BV-2 microglial cells. This absence of cytotoxicity of food-derived peptides has been extensively reported for several sources. The lack of cytotoxicity can be explained because the test items are peptides derived from a bond cleavage process that did not modify the amino acids of the sequences. The protein hydrolysates in this study had a degree of hydrolysis of 30 and 60%, respectively, with a high content of amino acids such as arginine, aspartic acid, or asparagine [9]. Protein hydrolysates from other plants such as wheat (gluten) or lupinus have also been shown to not affect cell viability [26,27], thus indicating that plant peptides produced after enzymatic hydrolysis are not toxic at the cellular level.

Nitric oxide (NO) acts as a component of the signaling pathways that operate across cerebral blood vessels, neurons, and glial cells, behaving like a neurotransmitter at the brain level. Its excessive production has been related to the development of several neurodegenerative diseases [28,29], when acting as a pro-inflammatory mediator. Physiologically, NO is toxic due to excess NO production by *iNos*. The results indicated that HPHs exert a similar in vitro effect to the specific inhibitor MCC950, at the concentration tested, thus diminishing the NO release after inducing inflammation. These results support the antioxidant properties described for the peptides previously [9]. The amino acid content of the hydrolysates analyzed in this study (containing asparagine glutamate or arginine in abundant quantities) contribute to modulate the production of nitric oxide, due to their functional groups [30].

In line with these results, Lin et al. [31] described the effect of heating on the digestibility of hemp seed protein and bioactivity of hydrolysates obtained by simulated gastrointestinal digestion (pepsin-pancreatin). These authors carried out measurement of released nitric oxide (NO), TNF-α, and IL-6 in RAW 264.7 macrophages, as an indicator of anti-inflammatory activity exerted by the peptides. The heat treatment led to a less bioactive hydrolysate than the one obtained without the heat pre-treatment. The differences between these hydrolysates could be explained by the peptides release due to the conformation of the protein to be hydrolyzed and the amino acid content. In fact, Bollati et al. [32] investigated the intestinal trans-epithelial transport and antioxidant activity of two peptides from hemp, proven to exert in vitro antioxidant activity. Authors reported that these peptides could reduce the H_2_O_2_-induced oxidative stress in HepG2 cells, as well as modulate the Nrf-2 and iNOS pathways, supporting the evidence that the amino acid sequences released after enzymatic hydrolysis possess a structure that interacts with the biological system. The mechanisms by which peptides modulate different physiological process is complex and should take into consideration several parameters. Overall, scientific evidence suggests that hemp protein hydrolysates are an adequate source of peptides able to counteract the release of NO in different cell models.

One step further, in order to elucidate the mechanisms behind the effects, the anti-inflammatory effect exerted by the test items at the brain level was evaluated by measuring the gene expression of the cytokines *tnfα*, *Il1β*, *Il6*, and *Il10*. As indicated in the results, the largest decrease of the expression of *tnfα* with respect to the control was observed in the sample employing the hydrolysate obtained with Alcalase and Flavourzyme. This effect might be due to the release of shorter peptides during the hydrolysis with Flavourzyme, as exopeptidase [33], which will more likely enter into cells and exert the reported effects due to the lower molecular weight. Similar results were reported by Cian et al. [34], who reported anti-inflammatory activity related to IL-10 expression and TNF-α inhibition exerted by seaweed *Ulva* spp. hydrolyzed either with Purazyme + Flavourzyme and Alkaline protease-Protex 6L + Flavourzyme, in splenic macrophages and lymphocytes. The functional groups contained in hemp peptides and the structure of these compounds are able to modulate in different cell models the inflammatory response.

The treatment with HPH20A only or in combination led to a decrease at the same range (no significant differences); whereas when the hydrolysate HPH60A+15F was used in combination with the inhibitor, the decrease is comparable to the observed in the control, and not the observed in the hydrolysate (the largest decrease). Thus, it supported the hypothesis that the decrease is mainly occurring due to the peptides released in the enzymatic treatment with Flavourzyme (i.e., molecular weight of peptides mainly from 300 to 400 Da) [9]. Similarly to these results, Girgih et al. [35] subjected hemp protein to simulated gastrointestinal digestion (pepsin and pancreatin), to evaluate the antioxidant activity of the digests. In the report, these authors indicated that low molecular weight peptides exhibited the highest radical scavenging activity, which support to the oxidative stress mediator potential proved of the hemp peptides. Moreover, Mahbub et al. [36] reported that hemp seed protein hydrolysates reduced the production of reactive oxygen species, inflammatory cytokines IL-8, IL-12p70, and IL-1β, in line with the results reported in our study. Evidence suggests that low molecular weight peptides, probably due to their low steric hindrance [37], can exert this bioactivity in the cell models employed, by binding to specific receptors thanks to the functional groups of the amino acids.

These results in the cell model, however, provide only a first understanding of the bioactivity of peptides. Cell-based analysis may narrow the gap between the in vitro and in vivo studies, providing a higher-quality characterization on the potential effect these peptides might have. In order to explain the effects that these peptides could affect in the CNS, an evaluation of the effects on microglia was carried out.

In a physiological context, depending on the injury or stimulus that triggers their activation through PRRs such as Toll-type receptors (TLR-4) or NOD-like receptors, microglia can adopt two different polarized phenotypes with different functions: the M1 phenotype or classical activation and the M2 phenotype or alternative activation/acquired deactivation. In order to understand how peptides, affect microglia, gene expression of markers from both phenotypes was performed.

To this purpose, the effect of the HPHs on the gene expression of different markers of the polarization of the microglia toward the phenotype M1 (*Ccr7* and *iNos*) or M2 (*Arg1* and *Ym1*) was determined, so as to unravel the mechanisms by which peptides exert these modulatory effects on the parameters evaluated. M2 microglia is involved in the restoration of tissue homeostasis after the elimination of foreign bodies by phagocytosis, directly participating in the positive regulation of anti-inflammatory cytokines, in the negative regulation of proinflammatory cytokines, and in remyelination [38,39]. Consequently, in the face of a neuroinflammatory process, the promotion of microglial polarization toward an M2 phenotype could be crucial for protecting the CNS. This work suggested that hemp hydrolysates not only decrease the gene expression levels of M1 polarization markers such as *Ccr7* and *iNos*, but also promote the gene expression of M2 markers such as *Ym1* in BV-2 cells treated with LPS. Based on the statistically significant differences found in the assayed parameters, these results suggest that hemp peptides are able to interact at cellular level with the microglia, modifying the neuroinflammatory response in cell models. However, these results should be validated by means of in vivo studies where the actual physiological conditions are considered. These preliminary results (i.e., increase in Arg1 mRNA) suggest that hemp peptides promote microglia shift from M1 to M2. However, further studies where the effect of peptides on other M2-specific genes (e.g., TGF-β, IL-3, IL4, IGF-1, FGF, CSF−1, NGF, BDNF) and protein levels is studied are needed in order to derive biologically relevant conclusions about these compounds.

Finally, taking into consideration that inflammasomes activation is related to the aforementioned factors, and directly related to the regulation of immune system cells, it has been shown that its inhibition could improve neurological damage [18]. For that reason, the effect of hemp peptides on the gene expression was also evaluated, in order to provide a whole description of the potential of these compounds in the neuroinflammation process.

The gene expression of two inflammasome components (i.e., *Nlrp3* and *Asc*), as well as the gene expression of molecules whose expression or functionality is regulated by the activation of this inflammasome (i.e., *Casp1* and *Il18*) were evaluated. As shown in the results, exposure to LPS caused a significant increase in caspase-1 expression, whereas all the treatments significantly counteracted this increase. This is in line with the effects observed for hemp peptides, having an adequate molecular weight and physico-chemical properties to be able to interact with the cells and modify the response.

In addition, as seen for the other proinflammatory cytokines involved in the inflammasome, all the treatments also repressed the gene expression of *Il18* with respect to the control with LPS. No significant differences were found among treatments, in contrast to the effects observed for *Nlrp3*. It has been shown for instance, that whey metal-binding peptides decrease nitric oxide production and tumor necrosis factor α (TNF-α) at mRNA and protein levels by stimulated BV-2 microglia. Authors indicated that this is due to the hydrophobicity and specific sequences, and peptides were also able to suppress NF-kB pathway, showing high affinity to bind NF-kB p65, evaluated by molecular docking [40]. Hemp peptides, high in hydrophobic amino acids, could exert a similar effect in the cell model used in this study.

These results are aligned with currently published literature. For instance, Zhang et al. [41] recently reported the effects that foxtail millet protein hydrolysates have against experimental colitis in mice. These authors indicate that the oral administration reduced the serum LPS level, inhibited NF-κB phosphorylation, and reduced the levels of TNF-α and IL-6. In addition, the hydrolysate led to an inhibition of inflammasome activation and IL-1β expression through the NLRP3/ASC/caspase-1 pathway. Similarly, Healy et al. [42] also reported attenuated IL-1β secretion in adipose tissue, that can be linked to attenuation of NLRP3 inflammasome, due to supplementation with casein hydrolysate. These authors observed that the pretreatment during 48 h with the hydrolysate, reduced caspase-1 activity and decreased IL-1β secretion from J774.2 macrophages in vitro that were LPS primed (10 ng/mL) and stimulated with adenosine triphosphate. In a recent review from Aguchem et al. [43], authors aimed to elucidate the potential mechanisms of antioxidant and anti-inflammatory properties of hempseed proteins and peptides, declaring that the amount of evidence by far is promising, though further research with in vivo models is required.

The molecular mechanisms by which these peptides can alter gene expression have not been explored in this research. However, as recently reviewed by Khavinson et al. [44], short peptides can interact with the nucleosome, the histone proteins, and DNA after penetrating into the nuclei and nucleoli of cells. The interaction between the DNA strands and the peptides would imply sequence recognition in gene promoters, which have a direct impact in template-directed synthetic reactions, replication, transcription, and reparation. In addition, these peptides can also regulate the status of DNA methylation, activating or repressing genes.

Further studies should consider the use of animal models in order to potentially observe effects in vivo. However, these results provide a description and characterization of protein hydrolysates with high potential to exert a biological relevant effect on microglia. This study lacks characterization of the peptides sequences and hinders the accurate correlation of specific compounds with the effects. However, based on the knowledge of the treatments and the characteristics of the peptides, as described, an insight of the most potent candidates test item has been discussed. In addition, further studies employing transwell systems in order to address the bioavailability of peptides is needed.

## 5. Conclusions

Hemp protein hydrolysates obtained by enzymatic hydrolysis with Alcalase or Alcalase followed by Flavourzyme is suggested to exert a potential neuroprotective effect in vitro via inhibiting NLRP3 inflammasome signaling pathway. This effect has been described in murine BV-2 microglial cells subjected to lipopolysaccharide (LPS)-induced neuroinflammation, followed by the exposure to the protein hydrolysate during 24 h. Results showed suppression of the expression of proinflammatory cytokines such as *Tnfα*, *Il1β*, *Il6*, *Ccr7*, *iNos*, *Nlrp3*, *Asc*, *Casp1*, and *Il18* and induction of the expression of anti-inflammatory cytokines such as *Il10* and *Ym1* in microglial cells, thus favoring their polarization toward an anti-inflammatory M2 phenotype. This in vitro approach supports the idea of employing hemp protein hydrolysates for functional food, although in vivo studies regarding the fate of these peptides should be conducted, to ensure that these peptides can cross the blood–brain barrier and finally interact with brain microglia. Therefore, dietary supplementation with hemp protein hydrolysates might contribute to the prevention or inhibition of neurodegenerative disorders.

## Figures and Tables

**Table 1:** Sequence and GenBank accession numbers of oligonucleotides used in RT-qPCR.

Target	GenBank Accession Number	Forward Reverse	Sequence (5′→3′)
*Tnfα*	NM_000594	Forward Reverse	TCCTTCAGACACCCTCAACC AGGCCCCAGTTTGAATTCTT
*Il1β*	NM_000576	Forward Reverse	GGGCCTCAAGGAAAAGAATC TTCTGCTTGAGAGGTGCTGA
*Il6*	NM_000600	Forward Reverse	TACCCCCAGGAGAAGATTCC TTTTCTGCCAGTGCCTCTTT
*Il10*	NM_000572	Forward Reverse	GCCTAACATGCTTCGAGATC TGATGTCTGGGTCTTGGTTC
*Ccr7*	NM_007719.2	Forward Reverse	GTGTGCTTCTGCCAAGATGA CCACGAAGCAGATGACAGAA
*iNos*	NM_ 000625	Forward Reverse	ACCCAGACTTACCCCTTTGG GCCTGGGGTCTAGGAGAGAC
*Arg1*	NM_007482.3	Forward Reverse	CGCCTTTCTCAAAAGGACAG ACAGACCGTGGGTTCTTCAC
*Ym1*	NM_009892.3	Forward Reverse	ACTTTGATGGCCTCAACCTG AATGATTCCTGCTCCTGTGG
*Nlrp3*	NM_001359638.1	Forward Reverse	AAGCAACAGATGGAGACCGG CAAATTCCATCCGCAGCCAG
*Asc*	NM_023258.4	Forward Reverse	GTCTTAGGGGCGGAAACCAA CCGCGGTCACCTTTTACTCT
*Casp1*	NM_009807.2	Forward Reverse	ACTGACTGGGACCCTCAAGT AACTTGAGCTCCAACCCTCG
*Il18*	NM_001357221.1	Forward Reverse	TCGCAGCAGGGTTTTCTAGG ACGGGAGGGAGAAAGACTGA
*Hprt*	NM_001289746	Forward Reverse	ACCCCACGAAGTGTTGGATA AAGCAGATGGCCACAGAACT
*Gapdh*	NM_001289726	Forward Reverse	AACTTTGGCATTGTGGAAGG ACACATTGGGGGTAGGAACA

## Data Availability

The data presented in this study are unavailable due to privacy restrictions.

## Appendix A

**Table A1:** Chemical composition of hemp seeds, HPI, HPH20A, and HPH60A15F. Data, expressed as percentage in dry basis, are mean ± standard deviation of triplicates.

Nutrient (g/100 g Product)	Hemp Seeds	Hemp Protein Isolate (HPI)	Hemp Protein Hydrolysate: HPH20A	Hemp Protein Hydrolysate: HPH 60A + 15F
Proteins	24.2 ± 1.0	96.5 ± 0.9	83.0 ± 0.2	82.3 ± 0.0
Carbs	3.8 ± 0.8	0.0 ± 0.0	0.0 ± 0.0	0.0 ± 0.0
Fat	32.4 ± 0.6	---------	---------	---------
Humidity	4.5 ± 0.0	2.2 ± 0.0	8.8 ± 0.0	7.5 ± 0.3
Ash	5.1 ± 0.1	1.1 ± 0.1	8.1 ± 0.8	10.1 ± 0.3
Fiber	29.7 ± 1.4	0.0 ± 0.0	0.0 ± 0.0	0.0 ± 0.0
Polyphenols	0.4 ± 0.0	0.0 ± 0.0	0.0 ± 0.0	0.0 ± 0.0

**Table A2:** Amino acid profile of defatted hemp flour (DHF), HPI, HPH20A, and HPH60A15F by HPLC. Values were expressed as milligrams of amino acids by grams of protein, are the mean ± standard deviation of triplicates.

Amino Acids (mg/g Protein)	Hemp Protein Isolate (HPI)	Hemp Protein Hydrolysate: HPH20A	Hemp Protein Hydrolysate: HPH60A+15F
Aspartic acid + asparagine	151.7 ± 0.8	142.2 ± 2.3	146.1 ± 1.7
Glutamic acid + Glutamine	194.2 ± 1.7	191.9 ± 2.3	189.8 ± 3.6
Alanine	47.6 ± 0.4	43.4 ± 0.5	44.7 ± 1.0
Arginine	126.5 ± 0.9	131.0 ± 1.2	131.6 ± 1.6
Cysteine	11.7 ± 0.3	5.8 ± 0.3	6.6 ± 0.3
Glycine	40.1 ± 0.2	36.7 ± 0.4	36.9 ± 1.0
Histidine	28.6 ± 0.2	28.5 ± 0.1	27.1 ± 0.1
Isoleucine	23.8 ± 0.0	41.0 ± 0.4	39.2 ± 0.7
Leucine	62.9 ± 0.4	62.3 ± 0.5	64.6 ± 0.9
Lysine	29.2 ± 0.2	33.1 ± 0.5	33.4 ± 0.4
Methionine	17.9 ± 6.2	24.2 ± 0.0	18.5 ± 0.4
Phenylalanine + Tyrosine	78.7 ± 0.7	78.6 ± 0.7	80.3 ± 1.2
Proline	58.6 ± 10.7	45.4 ± 9.5	28.8 ± 0.4
Serine	59.6 ± 0.4	50.0 ± 0.4	54.1 ± 0.8
Threonine	34.1 ± 0.4	32.1 ± 0.2	34.4 ± 0.5
Tryptophan	11.9 ± 0.2	10.7 ± 0.0	11.1 ± 0.5
Valine	34.8 ± 1.1	43.4 ± 0.5	42.6 ± 1.0
